# Corrective steps during neonatal mask ventilation – a narrative review of the evidence behind the MR SOPA acronym

**DOI:** 10.1016/j.resplu.2026.101288

**Published:** 2026-03-12

**Authors:** Vincent D. Gaertner, Lukas P. Mileder, Laila Springer, Michael Wagner, Robyn Dvorsky, Christoph M. Rüegger, Maxi Kaufmann

**Affiliations:** a Division of Neonatology, Dr von Hauner Children’s Hospital, LMU Hospital, Ludwig-Maximilian-Universität, Munich, Germany; b Working Group “Postnatal Support of Neonatal Transition/Resuscitation”, Society for Neonatology and Paediatric Intensive Care Medicine, Berlin, Germany; c German Center for Lung Research (DZL), Comprehensive Pneumology Center Munich, Germany; d Division of Neonatology, Department of Paediatrics and Adolescent Medicine, Medical University of Graz, Graz, Austria; e Department of Neonatology, University Children’s Hospital Tübingen, Tübingen, Baden-Württemberg, Germany; f Division of Neonatology, Pediatric Intensive Care and Neuropaediatrics, Department of Paediatrics, Comprehensive Center for Paediatrics, Medical University of Vienna, Vienna, Austria; g Newborn Research, Department of Neonatology, University Hospital and University of Zürich, Zürich, Switzerland; h Division of Neonatology & Paediatric Intensive Care Medicine, Department of Paediatrics, University Hospital Carl Gustav Carus Dresden, Dresden, Germany

**Keywords:** Neonatal resuscitation, Postnatal stabilization, Non-invasive ventilation, Bag-and-mask ventilation, Endotracheal intubation, Suctioning, Facemask

## Abstract

The mnemonic “*MR SOPA*” (*M*ask adjustment, *R*epositioning head/airway, *S*uctioning, *O*pen mouth, *P*ressure increase and *A*lternative airway) facilitates remembering corrective steps when ventilation during neonatal resuscitation is inadequate. Here, we critically evaluate the scientific evidence for each step and appraise the usefulness of the mnemonic as a sequential guidance in airway management of newborn infants:

*Mask:* Size and placement are crucial to minimize mask leak and airway obstruction. Firm top and flexible edges may help form a better seal, while mask shape seems to be less important. Two-person holding technique may optimize applied tidal volumes.

*Repositioning:* A neutral head position in newborns may improve airway patency.

*Suctioning:* Suctioning should be reserved for infants with perceived airway obstruction to reduce vagal stimulation or tissue damage.

*Open the mouth/airway:* There is no data on opening the mouth *per se*. Airway maneuvers like chin lift and jaw thrust may improve airway patency.

*Pressure increase:* Despite weak evidence, increased PIP of ≥25 cmH_2_O may be necessary to overcome closed glottis and fluid-filled lungs in non-breathing infants but must be titrated carefully to preclude lung injury.

*Alternative airway:* Nasopharyngeal tubes and laryngeal masks are valid options when face mask ventilation fails. Endotracheal intubation remains the gold standard but should be reserved for experienced staff.

These statements are based on scarce and limited evidence, largely from preclinical or smaller clinical studies. There is no evidence for performing the MRSOPA steps in its original sequence. Thus, more rigorous studies are needed to substantiate nature, timing and order of the interventions.

## Introduction

Approximately 1.5 million neonates die on their first day of life, mostly in resource-limited settings.^1^ The major reason for mortality among term born infants is perinatal asphyxia which could be prevented in many cases by simple resuscitative measures such as adequate ventilation via bag-and-mask or a T-piece.^2^ While the helping babies breathe (HBB) algorithm helped in reducing mortality in resource-limited settings, it still remains a large contributor to the high under-5 mortality in multiple countries.^3^

The lung needs to transition from a liquid-filled to an air-filled organ over the first inflations in order to allow gas exchange in non-breathing infants. Consequently, positive pressure ventilation (PPV) remains the therapeutic cornerstone for apneic infants regardless of gestational age.^4^ Irrespective of situation and equipment, failure to adequately aerate and ventilate may result in mortality or long-term morbidity in all gestational age groups.^5^

Consequently, air leak and airway obstruction are impediments that need to be minimized during neonatal resuscitation. When ventilation is inadequate, corrective steps need to be performed in order to improve resuscitative efforts. The Neonatal Resuscitation Program has introduced the mnemonic “*MR SOPA*” to facilitate remembering important steps to improve PPV, including *M*ask adjustment, *R*epositioning of head/airway, *S*uctioning, *O*pening the mouth, *P*ressure increase and *A*lternative airway use.^6^ In this narrative review, we aim to describe and critically appraise the scientific evidence for each step contained in the MR SOPA acronym.

## Main part

### Mask leak and airway obstruction

Mask leak and airway obstruction are very common during neonatal resuscitation,^7^, ^8^ largely independent of the resuscitator’s experience and patients’ gestational age.^9^, ^10^, ^11^, ^12^, ^13^ Physiologically, while PEEP can be delivered even with mask leak present,^14^ variable mask leak will result in variable gas flow, corroborated by the fact that delivered tidal volumes in preterm infants range from close to 0 ml/kg to a damagingly high 30 ml/kg in the delivery room.^15^ Similarly, airway obstruction for anatomical (i.e. tongue obstructing the pharynx), physiological (i.e. a closed glottis and/or retained secretions in the airways), and iatrogenic reasons (i.e. excess pressure from the mask and/or flexing/overextending the neck) may contribute to inadequate ventilation.^16^ In order to ensure adequate air entry and simultaneously minimize potential harm, it may be important to reduce leak and obstruction, even if clinical data supporting this assumption are lacking.^17^

**Fig. 1:**
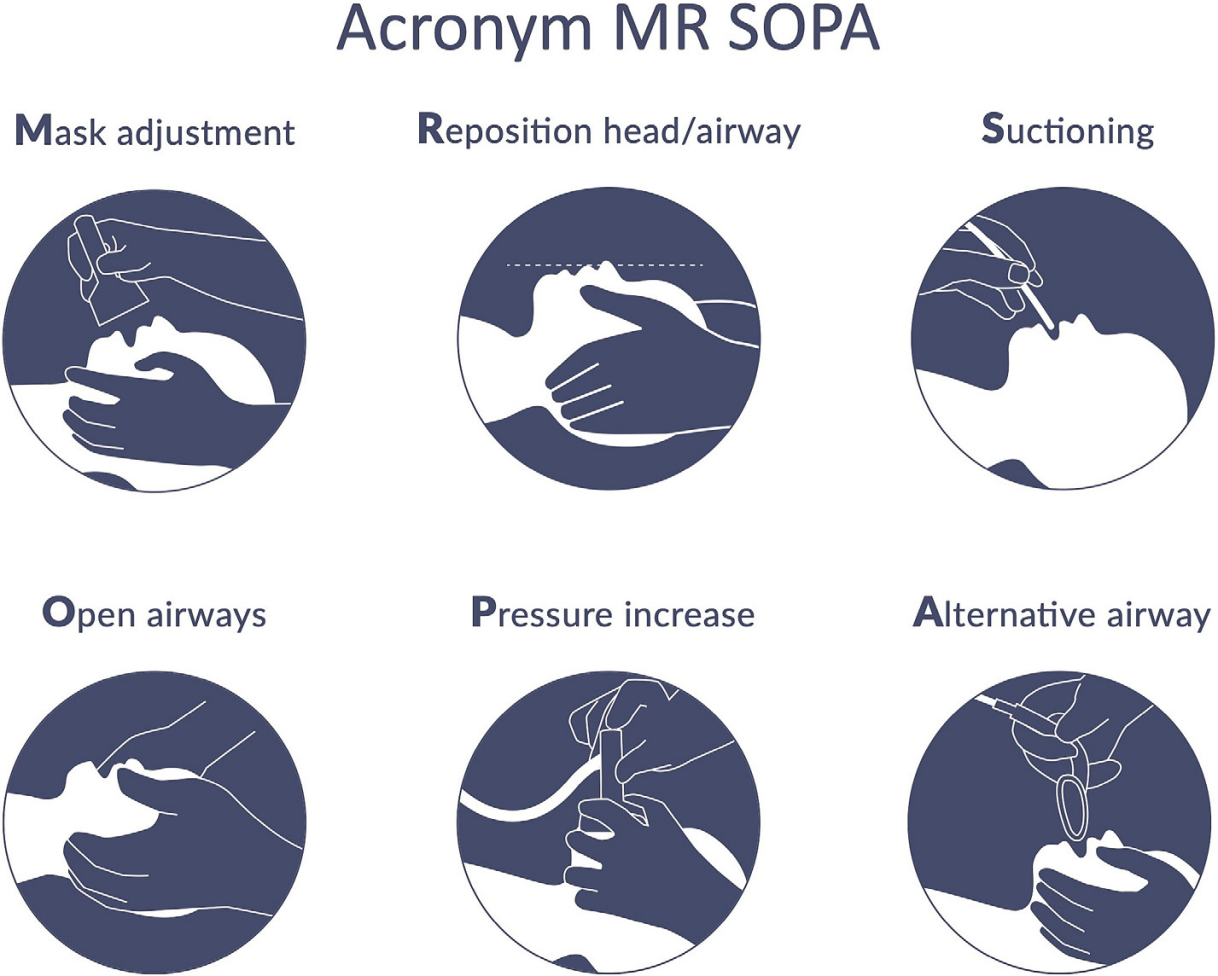
Visualization of the MR SOPA acronym.

The MR SOPA mnemonic may help consider steps to reduce leakage and obstruction. Its use is illustrated in Fig. 1, as well as in supplementary Video S1. Relevant papers were found by systematically searching OVID Medline, PubMed, Embase and Scopus and cross‐checking the reference lists of relevant articles (see supplementary Table S1 for search terms). Results were limited to papers that were published in English between inception of the respective database and September 30, 2025. All included studies are summarized in supplementary Table 2.

### Mask adjustment

Usually, ventilation is performed using a face mask during neonatal stabilization of preterm and term infants. Other interfaces may be used in specific situations or certain neonatal units for primary respiratory support. In all situations, however, the interface needs to fit the infant’s face well in order to minimize leak and/or obstruction and ensure adequate air entry into the lungs. In a recent observational study of 132 infants across all gestational ages, airway maneuvers were performed in over 75% of infants, and repositioning of the face mask was the most commonly performed maneuver (57%).^18^

(1) Mask size and placement of the mask

The mask should cover the infant’s mouth and nose but not supersede their chin or cover their eyes and accordingly, mask size may need to be individualized to the infant.^13^ The mask needs to be placed centrally on the face (as opposed to lateralized) to preclude mask leak and airway obstruction. Recently, O’Shea and colleagues found that even the smallest size of some face mask brands is too large for many extremely preterm infants.^19^ Based on photographs, masks of 35 mm diameter are suitable for infants below 29 weeks' PMA and masks of 42 mm diameter are suitable for infants 27–33 weeks' PMA.^19^ However, a subsequent clinical trial randomizing very preterm infants to PPV with either a standard size mask with a diameter of 50 mm or a “fitting” smaller mask of either 35 mm or 42 mm based on their gestational age found no difference in terms of mask leakage or minimal oxygen saturation during PPV.^20^ Of note, mask leak was large (approximately 40%) in both groups, and the poor general seal may have contributed to the lack of significant differences between groups.

(2) Mask type

There are differently shaped face masks, some are in triangular shape (“anatomical”), others round and they are both used in different neonatal units. There is no evidence to support either of these shapes,^12^ with similar mask leakage between anatomical and round face masks, even when the anatomical mask was applied in 90° or 180° rotation.^21^ Unsurprisingly, in anatomical masks with an inflatable cushion, failure to appropriately inflate the cushion was associated with increased mask leak (52% vs. 27%) in a neonatal manikin.^21^ The applied force to the face was greater using anatomical face masks than round ones.^22^

Different physical properties of masks have been investigated during face mask ventilation in smaller studies: A study from 2008 compared two round face masks in a manikin study and did not find significant differences in mask leakage between the masks manufactured from Laerdal and Fisher & Paykel, respectively.^13^ In a randomized controlled trial comparing two soft round face masks during delivery room stabilization of very preterm infants, there was no difference in mask leak or delivered tidal volumes.^23^ Recently, a Laerdal face mask with a so-called snap-fit feature including a thicker top surface and a more pliable bottom decreased mask leak in a randomized manikin study from 37% to 14% when compared to the standard Laerdal newborn face mask.^24^

As an adequate seal is important, a mask was developed with a suctioning outer rim which was supposed to reduce leakage. While in a manikin model, this suction mask did reduce mask leak,^25^ mask leak was not reduced in a clinical setting but the mask compromised applied pressures^26^ and flow.^27^

(3) Mask holding technique

In a recent manikin study the investigators compared different techniques of holding the mask: the “stem hold” (where only the stem of the mask is held), the “two-point top hold” (where the mask is pressed to the infant’s face using the thumb and index finger), and the “OK rim hold” (where the entire rim is held with thumb and index finger thereby forming a C).^28^ The “two-point top hold” technique reliably reduced leak, whereas the “stem hold” was associated with the largest leak.^28^ In a subsequent randomized crossover study comparing three types of mask hold in a manikin (“two-point”, “two-handed” and “spider”), there was no difference in mask leak between groups.^29^

Usually, the face mask is held on the neonate's face with one hand, while the other hand delivers PPV. In a two-person technique, one caregiver holds the face mask with both hands, while another caregiver delivers PPV. In newborn infants, the two-handed technique has been shown to reliably reduce mask leak.^10^, ^30^, ^31^ A study by Tracy et al., examining over 20,000 inflations, demonstrated a 50% reduction in mask leak using the two-handed technique compared to the one-handed technique (5% vs. 12%).^30^ In a randomized cross-over simulation study, there were increased expiratory tidal volumes (9.7 mL/kg vs. 8.4 mL/kg) and reduced mask leak with the two-person technique. Additionally, there was increased attention to correct application of bag-mask ventilation.^32^ However, there is conflicting evidence from other studies regarding the magnitude of tidal volumes but there was consistently decreased mask leak with the two-person hold.^33^, ^34^

(4) Effect of instruction and training

Instruction and demonstration of how to ideally place and hold a face mask reduced mask leak by almost 25% in clinical staff in a pre-post manikin study.^28^ Similarly, after training in mask handling, mask leak decreased from 70% to 10% in a term manikin study with clinical staff while airway obstruction remained fairly similar.^35^

### Reposition head/airway

Airway obstruction is a common obstacle to effective mask ventilation,^8^, ^36^ and it is more frequently associated with clinical deterioration and the need for intubation in comparison with leak.^36^ Textbooks on neonatal resuscitation primarily describe a hyperflexed or hyperextended head position during mask ventilation as one of the reasons for airway obstruction.^6^

Newborns differ from older children and adults primarily in their relatively large occiput with the consequent tendency to flex the head slightly when lying supine. Also, their relatively large tongue allows for bulging in the oropharynx.^16^

Thus, the European Resuscitation Council (ERC) guideline recommends the so-called ‘sniffing’ or neutral position for newborns.^4^ The neck should not be flexed or extended to any significant degree and the infant's face should directly face the ceiling.

The evidence behind this recommendation is weak. In an MRI study in sedated children between 0 and 4 months, a patent airway was observed with an angle of 144–150° between occiput-opisthion-cervical spine.^37^ Postmortem, neck flexion raised the closing pressure, making the airway more susceptible to collapse.^38^ In addition, there is one study in 17 preterm infants ready for discharge who were placed in a car seat with and without a foam insert that provided a slot for the back of the infants' head allowing the infant to maintain the head in a neutral position. During neutral head position infants had a larger upper airway space measured by the respiration timed inspiratory radiographs and less desaturation and bradycardia.^39^ In a randomized controlled trial in 78 term infants, this insert was associated with less obstructive apnea and fewer severe desaturation events but there was no difference in the overall rate of desaturations.^40^

Haase et al. used fixed facial landmarks to define a reproducible angle and observed high interobserver correlations.^41^ In a prospective study using a sagittal positioned digital camera during mask ventilation in 45 preterm and term infants, the authors demonstrated an association between airway obstruction detected using a respiratory function monitor and a hyperextended head position >90°.^42^

Future clinical studies are needed to further confirm that the neutral position of the head is superior to the flexed or extended head position to avoid airway obstruction during mask ventilation. Definition of head position should be as simple and reproducible as possible to be implemented in clinical practice.

### Suctioning

Current ERC guidelines clearly state that “there is no evidence that normal lung fluid and secretions cause obstruction”,^4^ which is the reason why routine suctioning of the upper oro- and nasopharyngeal airway after birth is not recommended any more, even in the presence of meconium-stained amniotic fluid.^43^ The ERC recommends considering inspection of the upper airway and suctioning under direct vision only in case of ineffective initial attempts at aeration and ventilation.^4^

Hypoxemia, bradycardia, and lower Apgar scores have been reported after oro- and/or nasopharyngeal suctioning, possibly due to vagal stimulation.^44^, ^45^, ^46^ More severe complications include longer time until reaching normoxia,^44^, ^47^ pharyngo-esophageal perforation,^48^ cardiac arrhythmias, and apnea.^49^ In addition, suctioning may consume significant time,^50^ which could cause a delay in initiating PPV in apneic neonates.^51^

Despite these clear and restrictive recommendations and well-known potential complications of upper airway suctioning, it is one of the procedures performed most often during postnatal stabilization and resuscitation,^46^ especially in preterm neonates.^52^ In a study by Berisha et al., 53% of preterm and term neonates requiring PPV had their airway suctioned, with airway suctioning occurring before initial PPV in 27%.^53^ In a multi-center analysis of 74,183 preterm and term neonates requiring delivery room interventions, Eckart et al. found that airway suctioning was the third-most applied intervention, used in 55% of neonates.^54^ This may be due to the long-standing history of routine airway clearance after birth.

However, even if postnatal airway suctioning is only required in a low number of delivery room resuscitations, clearing the airway in such cases of obstruction due to secretions is an important intervention. In a video-based analysis of postnatal stabilization and resuscitation of depressed neonates, airway suctioning improved spontaneous breathing efforts in 72% of term neonates.^55^ Interestingly, such an effect was not observed in suctioned preterm neonates, possibly due to the longer and deeper suction efforts in this cohort.^55^

Typical devices for upper airway suctioning include thin, flexible plastic catheters, bulb suction devices, and the rigid Yankauer sucker.^4^ In a comparison of these different suction devices, 6- and 8-French flexible catheters were less effective in suctioning saline, while the pediatric Yankauer catheter and bulb syringe were superior in suctioning simulated meconium.^56^ However, suction bulbs may generate significant negative pressures, which could increase the risk of airway injuries especially in preterm neonates.^57^ Also, wiping of mouth and nose with a towel has been shown to be equivalent to oro-nasopharyngeal suctioning after birth, at least in stable preterm and term neonates being mainly delivered from clear amniotic fluid.^58^

In sum, oro-/nasopharyngeal suctioning remains an important intervention in selected infants suffering from severe airway obstruction due to secretions. However, it should be used cautiously, particularly in preterm infants to prevent lung volume loss and other potential adverse effects.

### Open the mouth

In non-breathing infants, opening the mouth is a prerequisite for successful ventilation. However, there is no evidence supporting or refuting this maneuver. Thus, we will also discuss maneuvers for opening the airway in general and to support spontaneous breathing.

Airway maneuvers such as the jaw thrust (Esmarch maneuver) and chin lift are recommended to achieve upper airway patency.^59^ Both jaw thrust and chin lift improved the glottis view compared to the neutral position during fiberoptic bronchoscopy in children.^60^ MRI studies have shown an increase in the diameter of the pharynx during chin lift,^61^ but not in all patients,^62^ and this was not always associated with improved ventilation.^63^ The jaw thrust maneuver also increased the pharyngeal diameter,^64^ and increased tidal volume, minute ventilation, and inspiratory and expiratory peak flow rates during elective fiberoptic bronchoscopy.^65^ Both, jaw thrust and the application of continuous positive airway pressure promoted glottic opening and reduction of stridor scores.^66^

Lateral body positioning may enlarge the upper airway (narrowest cross-sectional area, airway length, and airway volume) in sedated children,^67^, ^68^ but clinical utility is unclear.

Improving spontaneous breathing efforts is crucial for an open airway. Means to stimulate spontaneous breathing include tactile stimulation and an optimal balance of oxygen and carbon dioxide. Tactile stimulation may increase spontaneous breathing efforts, even during face mask ventilation,^69^, ^70^, ^71^ as well as oxygenation (88% vs 82%).^72^ Hypoxia can lead to apnea but free oxygen radicals may cause neurological damage, and the optimal initial oxygen concentration for newborn infants remains a subject of current debate.

In sum, it is paramount to increase spontaneous breathing efforts during neonatal stabilization, particularly in preterm infants. In infants requiring non-invasive respiratory support, the chin lift and jaw thrust maneuvers may be helpful to open the airways but randomized studies are lacking.

### Pressure increase

A key contributor to failed resuscitation are insufficient peak inspiratory pressures (PIP), which is particularly critical in the presence of reduced lung compliance, fluid-filled alveoli, or increased airway resistance.^73^ Although there are no clinical trials directly assessing the effectiveness of increasing PIP during postnatal resuscitation, the approach is grounded in fundamental respiratory physiology and supported by clinical experience.^74^

Several studies have shown that non-invasive PPV is often ineffective in establishing lung aeration in the absence of spontaneous respiratory efforts.^8^, ^36^ Recent evidence indicates that, in preterm infants, the vocal cords remain closed during apnea, between breaths, and during breath holds, thereby limiting the effectiveness of pressure transmission during PPV.^75^ Likely, infants close their glottis to facilitate lung aeration via pendelluft mechanisms.^76^ Supporting and stimulating spontaneous respirations not only promotes laryngeal opening but also represents the most physiologic and least injurious approach to respiratory support in the delivery room.^71^

However, in infants not breathing spontaneously, the provision of non-invasive intermittent positive pressure ventilation (NIPPV) is required. In term infants, historical data suggest that inflation pressures of up to 30 cm H_2_O are generally sufficient to aerate the lungs.^77^ Effective resuscitation requires titrating PIP based on clinical response, with the goal of achieving adequate lung aeration while minimizing the risk of injury. Most neonatal resuscitation algorithms recommend incremental increases in PIP – typically by 5 cm H_2_O.^4^ Physiological measurements in asphyxiated infants have demonstrated that PIPs of 30–40 cm H_2_O held for one second generate mean tidal volumes of 5 mL/kg.^78^ This volume is less than half of that typically generated by spontaneously breathing newborns during the early respiratory transition at birth, suggesting that higher inflating pressures may be necessary to establish functional residual capacity initially.^79^, ^80^, ^81^ Similar peak pressures generated higher tidal volumes once the lung was inflated, suggesting that higher pressures may be needed in some non-breathing infants.^82^ A prospective study involving 821 near-term and term neonates who underwent bag-mask ventilation without PEEP in a low-resource setting reported a median required PIP of 37 cm H_2_O to achieve clinical stabilization.^83^ In preterm infants, effective chest wall expansion has been observed with PIPs ranging from 14 to 30 cm H_2_O.^84^

In the absence of clinical studies evaluating the effects of various PIP levels on lung aeration, gas exchange, or clinical outcomes, the adequacy of ventilation must be assessed by clinical signs, including visible chest rise, heart rate response, and peripheral oxygen saturation.

While increasing PIP may be life-saving, excessive pressures pose substantial risks, including barotrauma, pneumothorax, volutrauma, and hemodynamic instability.^85^, ^86^ Preterm infants are especially vulnerable due to their immature lung architecture and fragile vasculature. In addition, high tidal volumes delivered during resuscitation have been associated with an increased risk of brain injury in this population.^87^ Even with standardized PIP settings, there was high variability in tidal volume delivery, underscoring the potential harm of uncontrolled pressure escalation.^88^ Clinicians should be vigilant in adjusting PIP as lung compliance improves during resuscitation. Failure to reduce inflating pressures appropriately may result in excessive tidal volumes.

Future research should prioritize individualized pressure levels that incorporate gestational age, real-time assessment of pulmonary function, and continuous feedback from monitoring tools.

### Alternative airway

Using a face mask is, by design, associated with increased dead space and a concomitantly slower time to achieve a set oxygen concentration.^89^ Also, application of a face mask after birth may be associated with apnea and bradycardia.^90^, ^91^

In infants who were unresponsive to face mask ventilation during neonatal transition, placing a nasopharyngeal tube increased lung volume and was associated with an improvement in vital parameters.^92^ Thus, a nasopharyngeal tube may be a good alternative in infants non-responsive to initial face mask ventilation in order to prevent intubation. This may be particularly important in extremely preterm infants, because other devices may not be suitable for this patient collective. There is no data on using an oropharyngeal airway after failure of facemask ventilation.

Laryngeal mask airways (LMA) are rather easy to use and can help establish a secure airway even in situations with a difficult airway. However, in the neonatal population, they are currently not routinely used. Of note, LMA are only available for infants above 1000–1500 g birth weight, depending on the manufacturer. Nonetheless, there are several case reports indicating that using LMA in infants as small as 800 g may be feasible.^93^ While most healthcare professionals consider the LMA as an alternative airway if mask ventilation is insufficient, most have very limited experience with the LMA.^94^ In meta-analyses, there were no clinically significant differences between using LMA or endotracheal tubes in insertion time or failure to correctly insert the device and more importantly, there was no difference in deaths or HIE events.^95^, ^96^ Thus, there is no clear benefit of an LMA compared with endotracheal intubation in infants failing primary ventilation but it may help circumventing invasive ventilation in some infants and may also be an alternative for inexperienced providers.

Endotracheal intubation currently remains the gold standard to establish a secure airway and guarantee adequate lung ventilation. However, there are adverse effects associated with intubation and mechanical ventilation, and the skill requires ample training to achieve procedural competence. Only about a fourth of all providers can intubate at the first attempt with three fourths requiring more than one attempt,^97^ especially among inexperienced providers.^98^ Repeated intubation attempts significantly increase the occurrence of adverse events, such as bradycardia and desaturation.^97^ Therefore, this procedure should be reserved for specialists.

### Sequence of MR SOPA

MR SOPA was initially designed as a series of sequential steps. There is no study investigating the use of the entire sequence until improvement of airway management. In contrast, most studies investigated single parts of the mnemonic. In a small observational study, the most common combination was M/R followed by S/O. In this study, interventions were sometimes associated with reduced mask leak and improved tidal volume but excessive tidal volumes were also noted. Also, obstruction was not resolved after most interventions.^99^ Thus, it is unclear whether these interventions cause more benefit or more harm to the infant. It is crucial that the clinician does not blindly follow a sequence of steps but rather follows the subtle signs from infant and monitoring devices.

While there is physiological rationale for the MR SOPA interventions in certain infants, there is no data underlining the performance of this sequence as opposed to a different sequence. There is the largest and most reliable amount of evidence surrounding alternative airways, then regarding mask placement, repositioning, opening mouth (and airways) and pressure increase, and finally, there is least evidence supporting routine suctioning. Clinicians may need to keep this in mind when trying to improve airway management in neonates.

## Discussion/conclusion

This narrative review highlights the physiology around the different aspects of the MR SOPA mnemonic to improve non-invasive ventilation of newborn infants immediately after birth and summarizes the available evidence. After decades of research, most of the evidence surrounding these different factors is observational or even anecdotal and only in some instances based on smaller randomized controlled trials. Still, the mnemonic may have its value in helping to remember potential steps to improve airway management, although the sequence may need to be adapted to each infant individually.

In infants who are non-responsive to face mask ventilation, adjustment of the face mask, repositioning of the head, suctioning, opening the mouth and upper airways, and pressure increase are measures which are physiologically sensible, but may be limited to selected infants. There is likely no necessity to perform all single, corrective steps in a cascade of interventions but rather it remains critical to clearly define the underlying problem and target the actions to the specific infant. Suctioning in particular should be performed with caution, as loss of positive distending pressure should be avoided during non-invasive ventilation after birth in very preterm infants. Additionally, tactile stimulation may assist in improving spontaneous breathing efforts in non-vigorous infants even during non-invasive ventilation,^71^ and the mnemonic may need to be adapted to include stimulation (and be called MR***S*** SOPA instead).

The MR SOPA mnemonic is an assisting tool, guiding the provider’s steps in a non-responsive infant immediately after birth. Individual parts are often performed unconsciously by healthcare providers before moving on to using an alternative airway to provide adequate oxygenation and decarboxylation. In our opinion, since there is no evidence of harm (except possibly for excessive suctioning), the mnemonic should be considered to help guiding clinicians during neonatal resuscitation. As high-quality evidence is largely still lacking for each individual component as well as the entire mnemonic, let alone its sequence, more research is needed to better understand the most effective steps to improve newborn ventilation. This could include further (larger) observational studies investigating physiological effects of each of the single parts of the mnemonic as well as randomized clinical trials, where ethically possible, to assess the direct effect of performing/not performing a certain measure.

## Ethics and patient consent

Not applicable.

## CRediT authorship contribution statement

**Vincent D. Gaertner:** Writing – original draft, Investigation. **Lukas P. Mileder:** Writing – review & editing, Investigation. **Laila Springer:** Writing – review & editing, Investigation. **Michael Wagner:** Writing – review & editing, Investigation. **Robyn Dvorsky:** Writing – review & editing, Investigation. **Christoph M. Rüegger:** Writing – review & editing, Investigation. **Maxi Kaufmann:** Writing – review & editing, Supervision, Investigation.

## Funding

VDG is funded by the German Research Foundation (DFG) via the Emmy-Noether-Program (Project number 547359845).

## Declaration of competing interest

VDG, LS, LPM, MBW, MK and CMR are members of the Working group “Neonatal transition/resuscitation”, Society for Neonatology and Pediatric Intensive Care Medicine, Berlin, Germany. There are no financial conflicts of interest to resolve.
