# Supplementary material for: Corrective steps during neonatal mask ventilation – a narrative review of the evidence behind the MR SOPA acronym
Source: Resusc Plus. 2026 Mar 12;29:101288. doi: 10.1016/j.resplu.2026.101288 (PMC13022702; doi:10.1016/j.resplu.2026.101288)
Supplement: Supplementary Table S1 [file mmc1.docx]

**Supplementary Table S1. Search strategy for each of the sections**

| Part of the acronym | Search strategy |
| --- | --- |
| Mask adjustment | (mask OR facemask OR adjust*) AND (newborn OR neonate OR infant OR preterm) |
| Reposition head/airway | (reposition* OR airway OR head) AND (newborn OR neonate OR infant OR preterm) |
| Suctioning | (suct* OR oropharyn* OR nasopharyn*) AND (newborn OR neonate OR infant OR preterm) |
| Open mouth/airway | (open* OR airway OR mouth) AND (newborn OR neonate OR infant OR preterm) |
| Pressure increase | (pressure OR increase OR NIPPV OR positive pressure) AND (newborn OR neonate OR infant OR preterm) |
| Alternative airway | (alternative airway OR airway OR LMA OR laryngeal mask OR nasopharyngeal tube OR endotrach* OR intubation) AND (newborn OR neonate OR infant OR preterm) |
