# Supplementary material for: Corrective steps during neonatal mask ventilation – a narrative review of the evidence behind the MR SOPA acronym
Source: Resusc Plus. 2026 Mar 12;29:101288. doi: 10.1016/j.resplu.2026.101288 (PMC13022702; doi:10.1016/j.resplu.2026.101288)
Supplement: Supplementary Table 2 [file mmc2.docx]

**Table 1.** Summary of studies included on mask adjustment.

| **Mask adjustment** | | | | |
| --- | --- | --- | --- | --- |
| **Study** | **Study type** | **Patient/study population** | **Main outcome measure** | **Main result(-s)** |
| Mileder LP et al. (2022)^18^ | Retrospective analysis of patients included in prospective observational studies | Preterm and term neonates | Frequency and effects of maneuvers to improve non-invasive respiratory support in neonates immediately after birth | Maneuvers to improve respiratory support were common during postnatal stabilization, and more frequent in preterm neonates |
| Wood FE et al. (2008)^13^ | Manikin study | Neonatal providers | Mask leak with the Laerdal round mask and a Fisher & Paykel mask | High average mask leak over 50%, no differences in mask leak between face masks |
| O’Shea JE et al. (2016)^19^ | Observational study | Preterm neonates at 24-33 weeks postmenstrual age | Measurements of the distance from the nasofrontal groove to the mental protuberance from standardized photos | Smallest face mask sizes may be too large for extreme preterm neonates |
| O'Currain E et al. (2019)^20^ | Parallel group, randomized controlled trial | Preterm infants ≤ 32 weeks of gestation | Mask leak either with a standard or a smaller face mask | No difference in mask leak |
| O'Donnell et al. (2005)^12^ | Manikin study | Neonatal providers | Flow, inspiratory tidal volume, airway pressure, and mask leak with the Laerdal and the Neopuff infant resuscitator, with round and anatomical face masks | No differences between face masks, but generally high mask leak and huge variation in tidal volumes |
| Deindl P et al. (2014)^21^ | Manikin study | Neonatal providers | Influence of mask type and mask position on effectiveness of positive pressure ventilation with ventilation bag | No difference in mask leak between anatomical and round face masks; no difference in mask leak based on positioning of the anatomical face mask; higher mask leak with empty air cushion rim |
| Hannan J et al. (2024)^22^ | Randomised cross-over simulation study | Neonatal providers | Applied force on the face and head during simulated mask ventilation with different face masks, devices and expertise levels | Applied force was greater with self-inflating bags than with T-piece devices and greater with anatomical than with round face masks; experts providers applied force more equally around the mask rim |
| Cheung D et al. (2015)^23^ | Randomized controlled trial | Preterm neonates <33 weeks of gestation | Mask leak between two round face masks (Fisher & Paykel and Laerdal) | No differences in mask leak and tidal volumes |
| Rafferty AR et al. (2018)^24^ | Manikin study | Neonatal providers | Mask leak, applied load, airway pressure and tidal volume with Laerdal Upright Resuscitator, standard Laerdal infant resuscitator and a T-piece resuscitator, either with the Laerdal snap-fit face mask or a standard round face mask | No difference in mask leak between resuscitation devices, but lower mask leak with the snap-fit mask in comparison to round face mask |
| Lorenz L et al. (2016)^25^ | Manikin study | Neonatal providers and students | Mask leak with a suction mask compared to a conventional face mask | Lower mask leak with the suction mask |
| Lorenz L et al. (2018)^26^ | Randomized controlled trial | Neonates >34 weeks of gestation | Effectiveness of positive pressure ventilation with a suction mask compared to a conventional face mask | No difference in mask leak, but lower peak inspiratory pressure and positive end-exspiratory pressure with the suction mask |
| Rüegger CM et al. (2019)^27^ | Manikin study | Information not provided | Effect of minimal and high mask leak during positive pressure ventilation with a suction mask | Peak inspiratory pressure and positive end-exspiratory pressure below set values were observed in presence of high mask leak |
| Wood FE et al. (2008)^28^ | Manikin study | Neonatal providers | Identification of a technique for face mask placement and hold with minimal mask leak | Lowest mask leak with “two-point top hold” |
| Wilson EV et al. (2014)^29^ | Manikin study | Neonatal providers | Comparison of mask leak with three methods of face mask hold | No difference in mask leak between the three techniques |
| Wood FE et al. (2011)^10^ | Manikin study | Neonatal providers | Mask leak and force applied to achieve mask seal either with a single-handed or a two-handed mask hold | Two-handed mask hold was associated with less mask leak but higher mean applied force |
| Tracy MB et al. (2011)^30^ | Manikin study | Neonatal providers | Mask leak during one- and two-person mask ventilation | Reduced mask leak with the two-person mask ventilation technique |
| Tracy MB et al. (2011)^31^ | Manikin study | Neonatal providers | Effectiveness of positive pressure ventilation either with ventilator-delivered mask ventilation or with hand-delivered mask ventilation using different devices | Least variation in delivered ventilation with ventilator-delivered mask ventilation |
| Bibl K et al. (2025)^32^ | Manikin study | Neonatal providers | Tidal volume, mask leak, inspiratory pressure, respiratory rate, and minute ventilation either with one- or two-person mask ventilation | Two-person ventilation technique was associated with increased tidal volume and decreased mask leak |
| Davidovic L et al. (2005)^33^ | Manikin study | Pediatric residents, emergency medicine residents, pediatric emergency department nurses, transport personnel, and paramedics | Mean tidal volume and peak pressures either with one- or two-person mask ventilation in a pediatric model | Higher mean tidal volume and peak pressures associated with the two-person mask ventilation technique |
| Shah D et al. (2023)^34^ | Pilot randomized controlled trial | Preterm neonates <30 weeks of gestation | Respiratory mechanics during one- and two-person mask ventilation | Higher mask leak with the one-person mask ventilation technique |
| Schilleman K et al. (2010)^35^ | Manikin study | Neonatal providers | Mask leak during positive pressure ventilation before training, after training in mask handling and three weeks later | Significant reduction of mask leak after the training, even at three weeks later |
| **Reposition head/airway** | | | | |
| **Study** | **Study type** | **Patient/study population** | **Main outcome measure** | **Main result(-s)** |
| Schmölzer GM et al. (2011)^36^ | Retrospective analysis of delivery room recordings | Preterm neonates <32 weeks of gestation | Prevalence of mask leak and airway obstruction during positive pressure ventilation | Obstruction (26%) and mask leak (51%) occurred frequently |
| Bhalala US et al. (2016)^37^ | Retrospective study of head and neck magnetic resonance imaging of neonates and infants | Neonates and infants until four months of age | Optimal head-tilt position for airway patency in neonates and young infants | >95% probability of patent airway at head-tilt angle of 144-150° |
| Wilson SL et al. (1980)^38^ | Post-mortem study | Deceased infants | Influence of transmural pressure and neck posture on upper airway patency | Neck flexion raised closing pressure, rendering the airway more susceptible to collapse |
| Tonkin SL et al. (2003)^39^ | Prospective pilot study | Preterm neonates approved for discharge | Effect of a foam insert in a car seat on upper airway space and continuous polygraphic recordings | Foam insert was associated with a larger upper airway space and reduced incidences of oxygen desaturation, bradycardia, and arousal |
| McIntosh CG et al. (2013)^40^ | Randomized controlled trial | Healthy term neonates | Effect of a foam insert in a car seat on upper airway obstruction and episodes of impaired oxygenation | Foam insert was associated with reduced rates of obstructive apnea, less severe desaturation events, and less time with oxygen saturation <85% |
| Haase B et al. (2021)^41^ | Prospective observational study | Near-term and term neonates | Reliability and reproducibility of angle measurements for neutral or sniffing position based on facial landmarks | Angle δ could be determined in all images, thus being reproducible and reliable |
| Haase B et al. (2023)^42^ | Observational pilot study | Preterm and term neonates | Head position during mask ventilation after birth, its variation between operators and potential association with airway obstruction | Hyperextended head position was associated with increased airway obstruction |
| **Suctioning** | | | | |
| **Study** | **Study type** | **Patient/study population** | **Main outcome measure** | **Main result(-s)** |
| Gungor S et al. (2006)^44^ | Randomized controlled trial | Healthy term neonates | Effect of routine oropharyngeal suctioning versus no suctioning on oxygen saturation, heart rate, and Apgar scores | Higher oxygen saturation levels, lower heart rate, and higher Apgar scores in the no-suction group |
| Purington C et al. (2023)^45^ | Retrospective observational study | Neonates ≥34 weeks of gestation | Potential suction-related heart rate responses in survivors and neonates who died within three days after birth | Suctioning was performed more frequently than recommended; in 13% of suction events, an arrhythmia or a brief/sustained >15% fall in heart rate was observed |
| Pike H et al. (2024)^46^ | Population-based observational study | Neonates ≥28 weeks of gestation | Incidence, sequence, timing, duration of and response to resuscitative interventions | High incidence of airway suctioning (35%), in 95% after initiation of positive pressure ventilation |
| Carrasco M et al. (1997)^47^ | Observational study with control group | Healthy term neonates | Effect of oronasopharyngeal suctioning on oxygen saturation | Lower oxygen saturation and longer time to reach saturations of 86% and 92% in the suction group |
| Sapin E et al. (2000)^48^ | Case series | Preterm neonates with iatrogenic pharyngo-esophageal perforation | Diagnostic and therapeutic approaches | Pharyngo-esophageal perforation was caused by “overenthusiastic routine postpartum suctioning or nasogastric tube insertion” in seven out of ten cases |
| Cordero L et al. (1971)^49^ | Observational study with control group | Term neonates | Heart rate either after repeated nasopharyngeal suctioning with a bulb syringe or with a nasogastric tube | Seven out of 46 neonates suctioned with a nasogastric tube developed severe cardiac arrhythmia, and five of them also became apneic |
| Konstantelos D et al. (2015)^50^ | Retrospective analysis of delivery room recordings | Preterm and term neonates | Incidence of suctioning in the delivery room and effect of suctioning on postnatal adaptation | 23% of term and 66% of preterm neonates were suctioned; suctioning did not influence heart rate and oxygen saturation in preterm neonates, but non-suctioned term neonates who also did not require respiratory support had higher oxygen saturation values |
| Ersdal HL et al. (2012)^51^ | Observational study | Preterm and term neonates | Time to initiation of spontaneous respiration or time until onset of breathing after stimulation/suctioning, or mask ventilation in apneic neonates | Risk for death or prolonged admission increased by 16% for every 30-second delay in initiating mask ventilation, and by 6% for every minute of mask ventilation |
| Konstantelos D et al. (2016)^52^ | Retrospective analysis of delivery room recordings | Preterm neonates | Process quality of delivery room management of preterm neonates | 71% were suctioned a median of two times |
| Berisha G et al. (2023)^53^ | Retrospective analysis of delivery room recordings | Preterm and term neonates | Effect of airway suctioning on muscle tone and skin color | Airway suctioning preceded the initiation of positive pressure ventilation in 27%; in 29.6 it stimulated a vigorous cry with improvements in muscle tone and skin color |
| Eckart F et al. (2025)^54^ | Multi-center observational study | Preterm and term neonates | Current practice of neonatal resuscitation and postnatal stabilization | Suctioning was the third-most applied intervention and used in 55% of supported neonates |
| Berisha G et al. (2023)^55^ | Prospective observational study of delivery room recordings | Preterm and term neonates | Postnatal airway suctioning and short-term outcomes in depressed neonates | Spontaneous breathing improved in 1.9% of preterm and 72.1% of term neonates |
| Zareen Z et al. (2013)^56^ | Bench study | N/A | Suctioning effectiveness of a solution of varying viscosity with flexible catheters, Yankauer suction tip and a bulb syringe | Yankauer suction tip and bulb syringe were more effective in suctioning simulated meconium |
| Alur P et al. (2012)^57^ | Bench study | Neonatal providers | Suction pressures with a bulb syringe | One out of six bulb syringes produced suction pressures below the recommended 100mmHg |
| Kelleher J et al. (2013)^58^ | Randomized equivalency trial | Neonates ≥35 weeks of gestation | Respiratory rate during the first 24 hours after birth either after gentle wiping of face, mouth, and nose with a towel or after suctioning of mouth and nostrils with a bulb syringe | Mean difference of one breath per minute |
| **Open the mouth/airway** | | | | |
| **Study** | **Study type** | **Patient/study population** | **Main outcome measure** | **Main result(-s)** |
| Umutoglu T et al. (2015)^59^ | Randomized controlled cross-over study | Patients aged between zero and 15 years | Influence of airway supporting maneuvers on glottis view | Jaw thrust, head tilt-chin lift and triple airway maneuver improved glottis view |
| Reber A et al. (1999)^60^ | Observational study | Spontaneously breathing, sedated children between two and eleven years of age | Effect of the chin lift maneuver on airway dimensions | Chin lift widened the entire pharyngeal airway |
| Reber A et al. (2001)^61^ | Observational study | Children between three and ten years of age with adenotonsillar hyperplasia | Effect of chin lift, jaw thrust – with or without continuous positive airway pressure – on upper airway dimensions and stridor | Chin lift or jaw thrust lifted the epiglottis; chin lift or jaw thrust in combination with continuous positive airway pressure reduced stridor |
| Bruppacher H et al. (2003)^63^ | Observational study | Anesthetized children between two and nine years of age with adenoidal hyperplasia | Comparative efficacy of chin lift, jaw thrust, and continuous positive airway pressure on airway patency and ventilation | Jaw thrust was the most effective maneuver to overcome airway obstruction |
| Hammer J et al. (2001)^65^ | Prospective, non-randomized study | Sedated infants | Impact of the jaw thrust maneuver or continuous positive airway pressure on tidal breathing | Jaw thrust increased tidal volume, minute ventilation, and peak tidal inspiratory and expiratory flows in all infants |
| Meier S et al. (2002)^66^ | Observational study | Anesthetized, spontaneously breathing children between two and nine years of age | Effect of chin lift or jaw thrust combined with continuous positive airway pressure on the size of the glottic opening and stridor score | Chin lift and jaw thrust combined with continuous positive airway pressure increased the glottic opening and decreased stridor |
| Li H et al. (2025)^67^ | Retrospective, observational study | Sedated, spontaneously breathing children under five years of age | Impact of lateral positioning on the upper airway morphology using MRI data | Lateral positioning enlarges the upper airway, especially narrowest cross-sectional area, anteroposterior diameter, airway volume, and airway length |
| Litman RS et al. (2005)^68^ | Observational study | Sedated, spontaneously breathing children between two and twelve years of age | Effect of lateral positioning on upper airway cross-sectional area and total upper airway volume | Lateral positioning increases upper airway total volume and widens upper airway, especially the region between tip of epiglottis and vocal cords |
| Gaertner VD et al. (2018)^70^ | Retrospective analysis of delivery room recordings | Preterm and term neonates | Investigation of tactile stimulation in the delivery room with regard to the side of application (drying, chest rub, back rub, foot flick), frequency, and infant response | Less mature infants are stimulated less frequently; most common responses were limb movement and crying/facial grimacing; crying was observed especially after truncal stimulation |
| Gaertner VD et al. (2022)^71^ | Secondary analysis of a prospective, randomized study of delivery room recordings | Neonates born $\geq$ 34 weeks of gestation | Effects of tactile stimulation on spontaneous breathing – number of spontaneous breaths, exhaled tidal volume, mask leak and obstruction - during PPV | During tactile stimulation number of spontaneous breaths and exhaled tidal volume increased, mask leak and obstructed inflations did not change |
| Dekker J et al. (2018)^72^ | Randomized controlled trial | Preterm infants born 27 – 32 weeks of gestation | Effect of repetitive stimulation on respiratory effort (egg. respiratory minute volume, tidal volumes, oxygenation) during first 4 min of life compared to standard tactile stimulation | No significant differences in respiratory minute volume, positive pressure administration or duration of ventilation; significant higher oxygen saturation and lower FiO2 during transport to NICU in intervention group |
| Heesters V et al. (2024)^75^ | Observational study | Preterm infants born < 30 weeks of gestation | Respiratory effort and percentage of closed vocal cords during ultrasound visualization, both during stabilization and one hour after birth | 40% of the included infants were apneic at birth, and in these infants the vocal cord remained closed almost the entire time during PPV; one hour later the vocal cords remained closed for 46% of the time |
| Gaertner VD et al. (2023)^76^ | Secondary analysis of a prospective randomized trial | Preterm infants born 26-32 weeks of gestation | Effect of lung volumes and ventilation distribution during different breath types after birth assessed by electrical impedance tomography | Identification of three different breath types: tidal breathing (44%), braking (50%)g, holding (6%)  End-expiratory lung volume increased only after holding breaths |
| **Pressure increase** | | | | |
| **Study** | **Study type** | **Patient/study population** | **Main outcome measure** | **Main result(-s)** |
| Hull D (1969) ^77^ | Prospective observational study | Asphyxiated neonates with Apgar score < 2 | Effect of positive pressure ventilation on lung volumes | Ventilating pressures of 30 cmH_2_0 provide adequate lung ventilation in most full-term infants |
| Boon AW et a. (1979)^78^ | Prospective observational study | Asphyxiated neonates born by cesarean section | Effect of positive pressure ventilation on tidal volume and functional respiratory capacity | Higher opening pressure required before lung expansion in asphyxiated neonates; gradual increase in tidal volume after initial lung expansion; faster development of FRC in spontaneous breathing infants |
| Milner et al. (1977)^79^ | Prospective observational study | Term neonates | Analysis of airway pressure during initial breaths and the effect of applied positive pressure on lung expansion | FRC initiated at first breath; Opening pressures >10 cmH₂O are rare in healthy term infants |
| Vyas et al. (1986)^80^ | Observational study | Term infants after vaginally delivery | Effect between inspiratory effort and FRC | **Significant correlation**between first inspiratory volume; inspiratory effort index and FRC; no significant correlation between first inspiratory pressure and FRC |
| Gaertner VD et al. (2026)^81^ | Prospective observational study | Healthy term infants after vaginally delivery | Effect of first breaths on EIT-derived lung function parameters | End-expiratory lung volume increased rapidly within the first breaths, tidal volumes were three-fold higher than post-transitional tidal volumes; peripheral lung under-ventilated initially |
| Bjorland PA et al. (2022)^82^ | Retrospective observational study | Term neonates | Analysis of inspiratory pressures and tidal volumes delivery by T-piece resuscitator | Median peak inflation pressure was 30 mbar; increased inflation times were associated with increased tidal volumes; inflation rates >40/min were associated with lower tidal volumes |
| Ersdal HL et al. (2020)^83^ | Observational study | Asphyxiated neonates born $\geq$ 36 weeks of gestation | Analysis of ventilation signals (pressure/flow), expired CO_2_ (ECO_2_) and heart rate during positive pressure ventilation performed using self-inflating bag | Median expired volumes (3.3 to 6.0 ml/kg), ECO2 (0.3 to 2.4%) and heart rate (109 to 138bpm) increased significantly within the first twenty PPVs, whereas the applied peak inflation pressure remained constant at a median of 37 mbar |
| Hird MF et al. (1991)^84^ | Observational study | Preterm neonates which required intubation during neonatal resuscitation | Determine inflation pressure for adequate chest expansion | Median needed inflation pressure was 22.8 cmH20; pressures $\leq$ 30 cmH2O were sufficient |
| Mian Q et al. (2019)^87^ | Observational study | Preterm infants born < 29 weeks of gestation | Association of tidal volumes during PPV with brain injury rates | Higher rates of brain injury in infants provided with tidal volumes > 6ml/kg compared to < 6ml/kg (51% vs. 13%) |
| Thomann J et al. (2022)^88^ | Secondary analysis of a prospective study | Neonates born $\geq$34 weeks of gestation | Analysis of tidal volumes during PPV using a respiratory function monitor | 36% of inflations had median tidal volumes in the target range (4–8 mL/kg), 42% above, and 10% below |
| **Alternative Airway** | | | | |
| **Study** | **Study type** | **Patient/study population** | **Main outcome measure** | **Main result(-s)** |
| Hishikawa K et al. (2017)^89^ | CPAP model study | N/A | Effect of face mask CPAP on system inner space volume | The evaluated dead space was 38.3ml |
| Gaertner VD et al. (2020)^90^ | Subgroup analysis of a prospective, randomised trial | Infants born >34 weeks of gestation | effect of first and subsequent facemask applications on spontaneous breaths | 11% infants stopped breathing after facemask application, higher rates of apnoea after first applications than in subsequent applications (29% vs 8%) |
| Kuypers KLAM et al. (2019)^91^ | Retrospective analysis of video recordings | Preterm born $\leq$  32 weeks of gestation | effect of face mask application on breathing | In 54% of infants, apnoea occurred at a median of 5 seconds after face mask application and lasted a median of 28 seconds; occurrence of apnoea was inversely associated with gestational age |
| Belting C et al. (2024) ^92^ | Observational study | Preterm infants non responsive to initial facemask ventilation | Changes on end-expiratory lung impedance (EELI), heart rate and SpO_2_/FiO_2_-ratio after the insertion of a rescue nasopharyngeal tube | Increase in end-expiratory lung impedance after insertion of a nasopharyngeal tube and improvement in heart rate |
| Brimacombe J (1999)^93^ | Case report | 24 week of gestation, male, 800g birth weight | N/A | Successful insertion of a size 1 laryngeal mask, despite manufacturer suggestions after failed intubation attempt |
| Trevisanuto et al. (2024)^94^ | Online, cross-sectional survey | Healthcare providers | Availability and experience with laryngeal masks in the delivery room | 26.1% delivery rooms lacked equipment; laryngeal masks considered after face mask or ET tube failure, mainly for infants with airway malformations; less effective than ET tube but less invasive |
| Singh N et al. (2022)^97^ | Retrospective analysis of the National Emergency Airway Registry for Neonates (NEAR4NEOS) | N/A | Association between tracheal intubation attempts and clinical adverse outcomes | 22% of intubations required ≥3 attempts; ≥2 attempts linked to higher risk of intubation-related events and severe oxygen desaturation |
| Evans P et al. (2021)^98^ | Multi-center cohort study | Neonatal-perinatal medicine fellows | progression toward neonatal intubation procedural competence during fellowship training | **77% of intubations were successful within two attempts; 45% of fellows achieved intubation competence (80% success rate within two attempts) during fellowship training; 8–46 intubation attempts were needed to achieve competence.** |
| Yang KC et al. (2020)^99^ | Prospective observational study | Preterm infants <32 weeks gestation | Effect of MR SOPA at mean exhaled tidal volume, mask leak and airway obstruction | 30 infants included (41 intervention periods); median 2 MRSOPA maneuvers per period, median duration 15 s; most common combination: Mask/Reposition + Suction/Open;  MRSOPA improved tidal volume in some inadequate cases, resolved mask leak in 69%, rarely resolved obstruction; bradycardia improved in 45% of cases, HR never worsened |
